# Potential Anti-Obesity Effect of Hazel Leaf Extract in Mice and Network Pharmacology of Selected Polyphenols

**DOI:** 10.3390/ph17101349

**Published:** 2024-10-09

**Authors:** Jiarui Zhao, Aikebaier Alimu, Yvmo Li, Zhi Lin, Jun Li, Xinhe Wang, Yuchen Wang, Guangfu Lv, He Lin, Zhe Lin

**Affiliations:** College of Pharmacy, Changchun University of Chinese Medicine, Changchun 130117, China; zjr0852@163.com (J.Z.); aikebaieralimu001@163.com (A.A.); 13596272257@163.com (Y.L.); linzhi1@jxutcm.edu.cn (Z.L.); 18224088551@163.com (J.L.); m17704303262_1@163.com (X.W.); wycscar@hotmail.com (Y.W.); message219@163.com (G.L.)

**Keywords:** hazel leaf, polyphenols, obesity, network pharmacology, gut microbiota, molecular docking

## Abstract

**Background:** Obesity is gradually becoming a widespread health problem, and treatment using natural compounds has seen an increasing trend. As a by-product of hazelnut, hazel leaf is usually disposed of as waste, but it is widely used in traditional and folk medicines around the world. **Aim of this study:** Based on previous studies, the effects of the regulation of lipid metabolism and the mechanism of hazel leaf polyphenol extraction obesity were investigated. **Methods:** In this study, a high-fat diet-fed mouse model of obesity and 3T3-L1 preadipocytes were established. The ameliorative effects of the hazel leaf polyphenol extract on obesity and the regulating lipid metabolisms were explored based on network pharmacology, gut microbiota, and molecular docking. **Results:** Network pharmacology showed that hazel leaf polyphenols may play a role by targeting key targets, including PPARγ, and regulating the PPAR signaling pathway. They significantly improved body weight gain, the liver index, and adiposity and lipid levels; regulated the gut microbiota and short-chain fatty acid contents; down-regulated the expression of lipid synthesis proteins SREBP1c, PPARγ, and C/EBP-α; and up-regulated the expression of p-AMPK in obese mice. They inhibited the differentiation of 3T3-L1 cells, and the expression of related proteins is consistent with the results in vivo. The molecular docking results indicated that gallic acid, quercetin-3-O-beta-D-glucopyranoside, quercetin, myricetin, and luteolin-7-O-glucoside in the hazel leaf polyphenol extract had strong binding activities with PPARγ, C/EBP-α, and AMPK. **Conclusions:** The results demonstrate that the hazel leaf polyphenol extract can improve obesity by regulating lipid metabolism, which provides a valuable basis for developing health products made from hazel leaf polyphenols in the future.

## 1. Introduction

According to the World Health Organization, the worldwide prevalence of obesity more than doubled between 1990 and 2022. In 2022, 2.5 billion adults (18 years and older) were classified as being overweight (43% of men and 44% of women); of these, 890 million were living with obesity. Additionally, 8% of children and adolescents were living with obesity (160 million young people) [1]. Obesity has become a global health problem and a major risk factor for type 2 diabetes, fatty liver, and cancer [2]. The underlying cause of obesity and overweight is an energy imbalance between calorie intake and consumption. Although the exact mechanism has not been elucidated, it is thought that the pathogenesis of obesity-related complications involves the abnormal production of adipokines byadipocytes [3].

Recently, the consumption of herbs, natural crude extracts, and natural compounds has been shown to be a promising, effective, and safe strategy for preventing obesity and related metabolic disorders [4]. *Paeonia lactiflora* root regulated lipolysis and thermogenesis, thus inhibiting obesity [5]. Rosemary inhibited lipid deposition and prevented inflammation and oxidative stress associated with obesity [6].Carambola leaf extracts reduced obesity induced by a high-fat diet (HFD) and improved hyperlipidemia, insulin resistance, and oxidative stress [7]. Mulberry leaf extract was able to inhibit weight gain and fat cell volume increase in HFD-induced obese mice [8]. Lychee polyphenols can reduce the activity of lipase and control the accumulation of adipose tissue in rats [9]. Green tea polyphenols significantly reduced the food intake of female mice, resulting in weight loss [10]. Polyphenols such as catechins contained in fermented green tea can decrease lipid content by inhibiting pancreatic lipase and increasing energy expenditure [11]. Ellagic acid in pomegranate peel can inhibit lipid synthesis by reducing the expression of FAS in 3T3-L1 adipocytes [12]. As one of the important natural products, polyphenols have received more attention in the prevention and treatment of obesity due to their good efficacy and safety.

Hazel leaf, a by-product of hazelnuts, is often disposed of as waste, but it is widely used in traditional and folk medicines around the world. In Iran, it is used as a liver tonic and also for hemorrhoids, varicose veins, phlebitis, and mild edema. In Sweden, hazel leaf and bark are used to treat pain, hemorrhoids, phlebitis, and other conditions. Galenic preparations of hazel leaves are also used to relieve ulcers and oropharyngeal infections and have mild anti-dysentery, anti-fungal, and scarring properties [13]. Studies have shown that hazel leaves are rich in polyphenols [14]. We previously extracted polyphenols from hazel leaves (*Corylus heterophylla* Fisch × *Corylus avellana* L.) and conducted a preliminary characterization of their composition, demonstrating their antioxidant and anti-inflammatory capabilities and preliminarily predicting the treatment of lipid metabolism and atherosclerotic-related diseases [15].

Based on these results, the present study combined network pharmacology and intestinal microbiota to preliminarily investigate the modulatory effects and mechanisms of a hazel leaf polyphenol extract (ZP) on a high-fat diet-induced obesity mouse model and 3T3-L1 cells, and molecular docking predicted the key active components of the hazel leaf polyphenol extract. This may provide a valuable basis for the exploitation and application of hazelnut leaves and their polyphenols as functional ingredients.

## 2. Results

### 2.1. Results of Network Pharmacology

**Figure 1:**
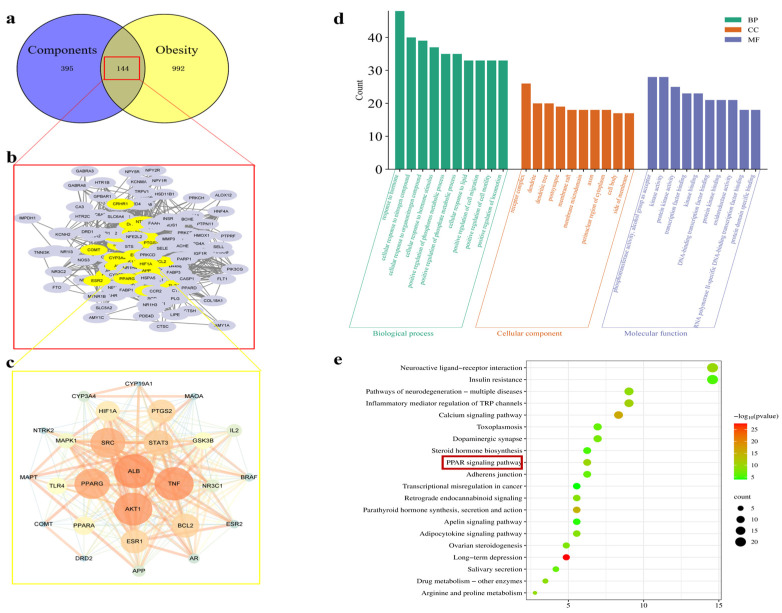
Analysis of network pharmacology. (**a**) Venn diagram analysis of targets of components with obesity. (**b**) A total of 144 intersecting targets. (**c**) The core target obtained from the screening (a larger circle represents a larger degree value of the target). (**d**) GO functional enrichment analysis. (**e**) KEGG pathway analysis.

The components of hazel leaf polyphenol extract (Table S1) were imported into the Swiss Target Prediction Database to obtain the targets, and a total of 539 targets were obtained after the screening and removal of duplicates (Table S2). The “obesity” targets mined from Gene Cards and other databases were screened and summarized, and duplicates were removed to obtain 1136 disease targets (Table S3). The intersection of the drug and disease targets was mapped, and 144 intersecting targets were obtained (Table S4). Twenty-seven core targets were obtained by Cytoscape analysis, among which the most important targets were ALB, TNF, AKT1, PPARG, and SRC. They are sorted by degree value in Figure 1a–c. GO analysis revealed 4983 biological processes (BPs), 377 cellular components (CCs), and 854 targets (CCs), with the top 10shown in Figure 1d. The KEGG-enriched pathways obtained include insulin resistance, the PPAR signaling pathway, and the apelin signaling pathway (Figure 1e). Among them, the PPAR signaling pathway is closely related to lipid metabolism and fat synthesis. Therefore, in vitro and in vivo experiments were further used to verify the above mechanisms.

### 2.2. Effects of ZP on Weight Gain, Food Intake, Liver Index, and Fat Content in Obese Mice

**Figure 2:**
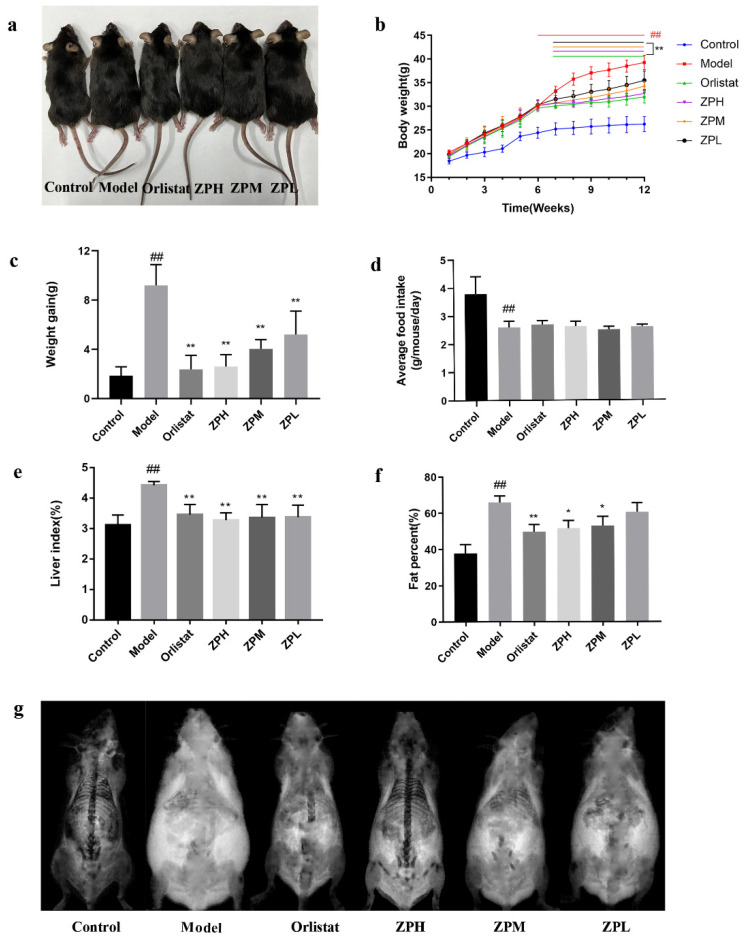
Effects of ZP on obese mice. (**a**) Morphological photographs of mice. (**b**) Mice body weight. (**c**) Weight gain. (**d**) Average food intake of mice. (**e**) Mice liver index. (**f**) Mice fat content. (**g**) Mice fat scan. Data are presented as means ± SD. ## *p* < 0.01 compared to control group. * *p* < 0.05, ** *p* < 0.01 compared to model group.

During the experiment, the weight of mice was recorded weekly, and the weight gain of the control group was stable and slow. Except for the control group, the weight of the other groups increased significantly over the six weeks. However, after the 7th week, the weight gain in the orlistat group and ZP groups began to decrease (Figure 2b). At the end of the experiment (12th week), the weight gain of mice in the orlistat group and ZP groups was significantly lower than that in the model group (*p* < 0.01) (Figure 2a,c). This indicated that hazel leaf polyphenol extract could slow down the mice’s weight gain on a high-fat diet. Meanwhile, the food intake of mice was recorded during the experiment. There was no significant difference in the food intake of the orlistat group and ZP groups compared with the model group (Figure 2d), indicating that hazel leaf polyphenol extract did not affect the food intake of mice and may not have exerted weight loss by suppressing appetite.

As shown in Figure 2e–g, compared with the control group, the liver index and fat content of mice in the model group were significantly increased (*p* < 0.01), while the liver index and fat content of mice in the orlistat group and ZP groups (500 and 250 mg/kg) were significantly lower than the model group (*p* < 0.05, *p* < 0.01). These results suggested that the high-fat diet caused the accumulation of lipids in liver tissue and increased body fat content, while hazel leaf polyphenol extract could attenuate the accumulation of lipids.

### 2.3. Effects of ZP on Blood Lipid Levels in Obese Mice

**Figure 3:**
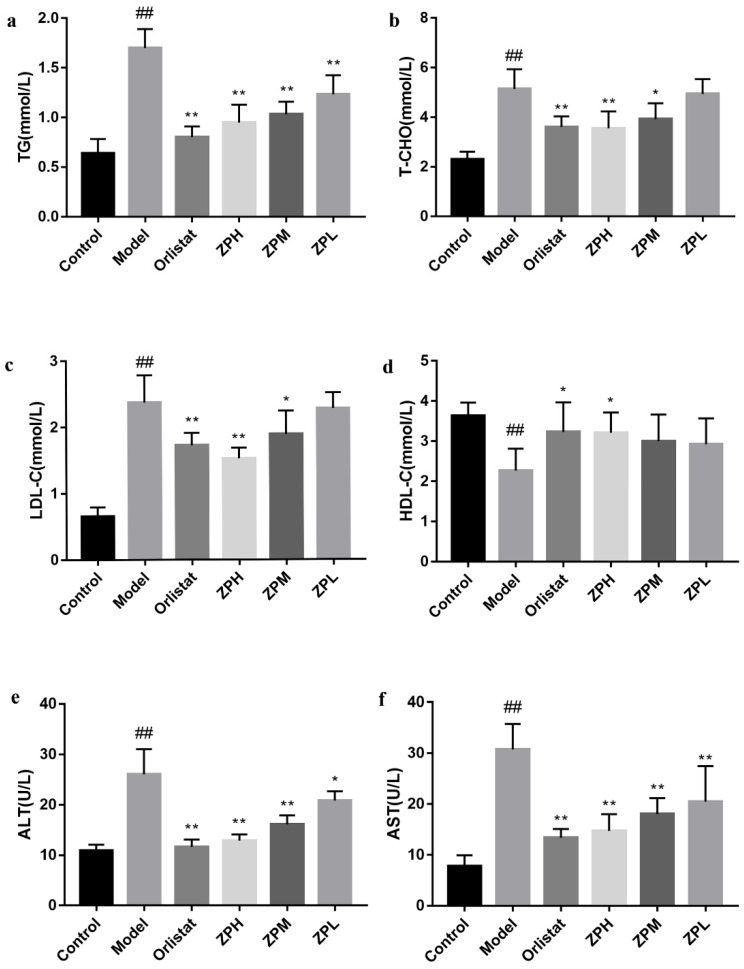
Effects of ZP on blood lipid levels, ALT, and AST in obese mice. (**a**) TG. (**b**) T-CHO. (**c**) LDL-C. (**d**) HDL-C. (**e**) ALT. (**f**) AST. Data are presented as means ± SD. ## *p* < 0.01 compared to control group. * *p* < 0.05, ** *p* < 0.01 compared to model group.

Blood lipid levels are one of the important indicators of metabolism in the body. As shown in Figure 3a–d, compared with the control group, the serum levels of TG, T-CHO, and LDL-C in the model group were significantly increased (*p* < 0.01), and the level of HDL-C was significantly reduced (*p* < 0.01).However, TG, T-CHO, and LDL-C levels were obviously lower in the ZP groups (500 and 250 mg/kg), and HDL-C levels were obviously higher in the ZPH group (500 mg/kg) compared to the model group (*p* < 0.05, *p* < 0.01).The blood lipid profile of other ZP dose groups was also improved, but there was no significant difference. These results revealed that hazel leaf polyphenol extract ameliorated the abnormal blood lipid levels of obese mice induced by a high-fat diet.

### 2.4. Effects of ZP on ALT and AST in Obese Mice

ALT and AST are commonly used as markers of liver damage, and serum ALT is the most sensitive indicator of liver fat accumulation. In this experiment, the serum levels of ALT and AST in the model group were significantly elevated compared to the control group (*p* < 0.01), demonstrating that obesity caused liver damage (Figure 3e,f). Compared with the model group, serum ALT and AST levels in ZP groups (500, 250, and 125 mg/kg) were significantly lower in a dose-dependent manner (*p* < 0.05, *p* < 0.01). This suggested that hazel leaf polyphenol extract could reduce liver lipid accumulation and alleviate liver damage in dietary obese mice.

### 2.5. Effects of ZP on Histological in Obese Mice

**Figure 4:**
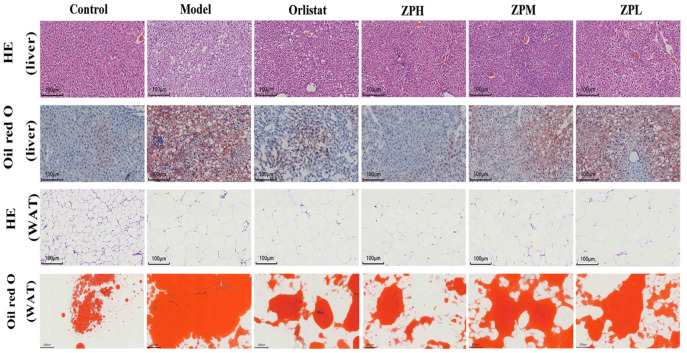
Representative images of liver and perirenal white fat (WAT) H&E staining and oil red O staining in different groups of mice. (HE staining of liver, WAT, and oil red O staining of liver scale bar, 100 µm; oil red O staining of WAT scale bar, 200 µm).

Mice liver HE and oil red staining showed that the liver tissue structure of mice in the control group was complete, the morphological size of hepatocytes was normal, the cell arrangement was uniform and compact, no steatosis was observed, and no lipid droplets appeared in liver cells. Compared with the control group, the liver cells in the model group were arranged and scattered, vacuoles of different sizes and numbers appeared, and lipid accumulation occurred in the liver to form lipid droplets. Compared with the model group, the orlistat group, ZPH group (500 mg/kg), ZPM group (250 mg/kg), and ZPL group (125 mg/kg) visibly reduced the number of cell vacuoles and lipid droplets (Figure 4).

Mice perirenal fat HE and oil red staining showed that the adipocytes in the control group were small in size, clear in morphology, and complete in structure, and the lipid droplet proportion was reduced. Compared with the control group, the fat cell volume in the model group was significantly increased, the cell size was uneven, the cell arrangement was irregular, and the lipid droplet proportion was larger. In the same field of view, the number of fat cells in the model group significantly decreased with the increase in size. Compared with the model group, the volume of adipocytes decreased to varying degrees in the orlistat group, ZPH group (500 mg/kg), ZPM group (250 mg/kg), and ZPL group (125 mg/kg), and the number of cells in the same field of view increased. The area of lipid droplets also clearly decreased. The results indicated that hazel leaf polyphenol extract alleviated the accumulation of lipid droplets in the liver and the increase in adipose tissue in obese mice.

### 2.6. Effects of ZP on Gut Microbes in Obese Mice

**Figure 5:**
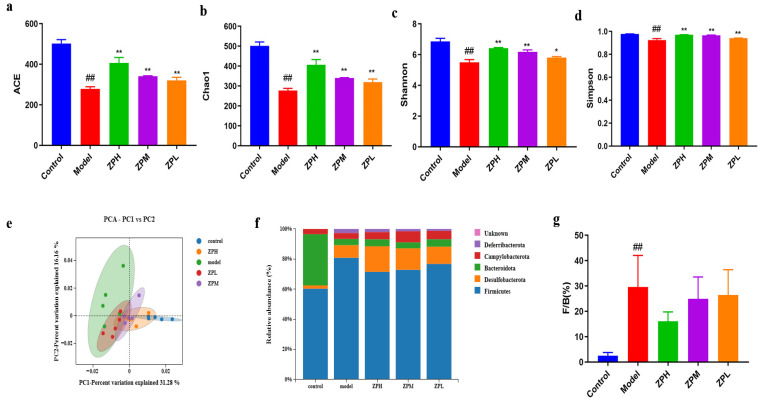
Effects of ZP on gut microbes in obese mice. (**a**) ACE. (**b**) Chao1. (**c**) Shannon. (**d**) Simpson. (**e**) Principal component analysis (PCA). (**f**) Phylum analysis. (**g**) F/B. ## *p* < 0.01 compared to control group. * *p* < 0.05, ** *p* < 0.01 compared to model group.

Alpha diversity reflects the species richness and species diversity of a single sample. The Ace andChao1 indices measure species richness, which is the number of species. Compared with the control group, the Ace index andChao1 index were decreased significantly in the model group(*p* < 0.01), while the indices of ZPH, ZPM, and ZPL groups were significantly higher than those of the model group(*p* < 0.01)in a dose-dependent manner(Figure 5a,b).The results showed that the gut microbes were decreased in high-fat diet mice and increased after hazel leaf polyphenol extract intervention. The Shannon index and Simpson index are used to measure species diversity and are influenced by species richness and species uniformity in the sample community. Compared with the control group, the Shannon index and Simpson index were significantly reduced in the model group (*p* < 0.01), while the indices of the ZPH and ZPM groups (500 and 250 mg/kg) were significantly higher than those of the model group(*p* < 0.05, *p* < 0.01) (Figure 5c,d). The results showed that the high-fat diet led to a decrease in gut microbes species diversity in mice, which was restored by hazel leaf polyphenol extract.

Principal component analysis (PCA, β diversity analysis) can reflect the similarity of samples between different groups, and the closeness of two samples is positively correlated with the similarity of their composition. As shown in Figure 5e, the samples of the model group and the control group were completely separated, indicating that a high-fat diet caused great changes in the gut microbial composition of mice. However, the gut microbiota of mice with ZP treatment was more similar to that of the control group, and this trend was dose-dependent. These results indicated that hazel leaf polyphenol extracts regulated the gut microbial composition of obese mice to a normal level.

In this study, the phyla with an abundance ratio greater than 1% were analyzed. The dominant phyla were *Bacteroidetes*, *Firmicutes*, *Desulfobacterota*, *Campylobacterota*, and *Deferribacterota*, among which *Firmicutes* and *Bacteroidetes* accounted for the highest proportion [16]. The *Firmicutes* phylum proportion in the model group was higher than that in the control group, and the proportion of *Firmicutes* decreased after ZP intervention (Figure 5f). The *Firmicutes*/*Bacteroides* (F/B) value, as an important indicator used to measure intestinal homeostasis, is closely related to body lipid metabolism [17]. The relative abundance of *Firmicutes* and *Bacteroides* in the gut flora may be biomarkers indicating susceptibility to obesity. Compared to the gut microbiota of healthy individuals, obese individuals have higher F/B values. Studies have shown that an increase in F/B value is beneficial for energy absorption and fat storage, leading to weight gain and obesity [18]. Our results showed that the F/B value of the model group was significantly higher than that of the control group. The F/B values of ZPH and ZPM groups (500 and 250 mg/kg) were decreased significantly compared with the model group (Figure 5g). This suggested that hazel leaf polyphenol extract could affect the structure of the gut flora of obese mice at the phylum level, which is similar to previous studies [19].

**Figure 6:**
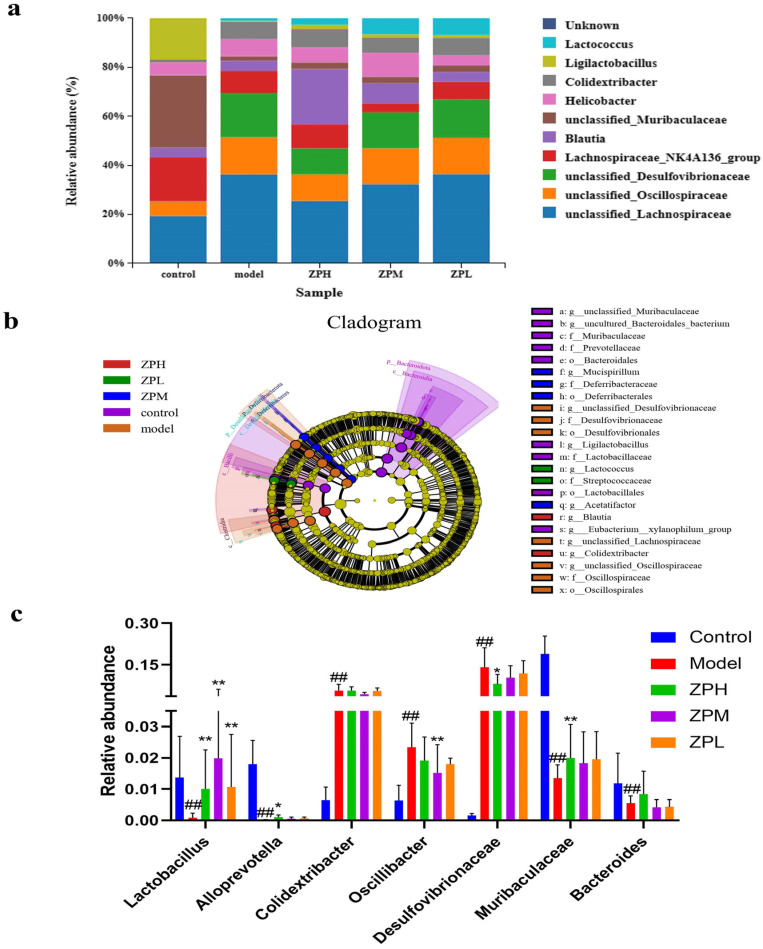
Effects of ZP on gut microbes at genus level in obese mice. (**a**) Genu analysis. (**b**) Cladogram. (**c**) Relative abundance. ## *p* < 0.01 compared to control group. * *p* < 0.05, ** *p* < 0.01 compared to model group.

At the genus level, compared with the control group, the relative abundance of unclassified*_Lachnospiraceae*, unclassified*_Oscillospiraceae*, and unclassified*_Desulfovibrionaceae* genus in the model group was apparently increased, while the relative abundance of *Ligilactobacillus* was apparently decreased. After the intervention of ZP, the relative abundance of unclassified*_Lachnospiraceae*, unclassified*_Oscillospiraceae,* and unclassified*_Desulfovibrionaceae* was decreased compared to the model group, while the relative abundance of *Ligilactobacillus* was increased (Figure 6a).

LEfSe analyses are used to assess the magnitude of colony abundance on differential effects, looking for statistically different species between groups. As can be seen in Figure 6b, different groups have different dominant genera. As shown in Figure 6c, compared with the control group, the relative abundance of *Lactobacillus*, *Alloprevotella*, *Muribacuaceae,* and *Bacteroides* in the model group was decreased significantly (*p* < 0.01), and the relative abundance of *Colidextribacter*, *Oscillibacter,* and *Desulfovibrionaceae* was increased significantly (*p* < 0.01). Compared with the model group, *Lactobacillus* and *Muribacuaceae* in the ZPH group (500 mg/kg) were significantly increased (*p* < 0.01), and *Desulfovibrionaceae* was significantly lower (*p* < 0.05). This part of the results coincided with the aforementioned changes in the diversity of the flora.

### 2.7. Effects of ZP on SCFAs Content in Obese Mice

**Figure 7:**
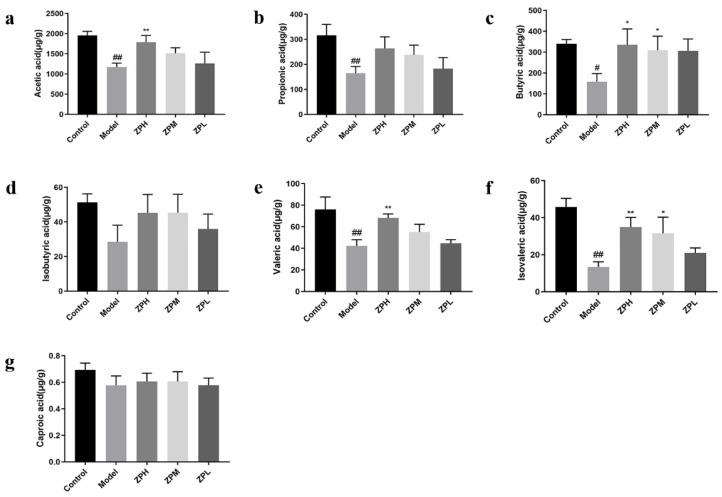
Effects of ZP on short-chain fatty acid content. (**a**) Acetic acid. (**b**) Propionic acid. (**c**) Butyric acid. (**d**) Isobutyric acid. (**e**) Valeric acid. (**f**) Isovaleric acid. (**g**) Caproic acid. # *p* < 0.05, ## *p* < 0.01 compared to control group. * *p* < 0.05, ** *p* < 0.01 compared to model group.

Short-chain fatty acids (SCFAs) are an energy source for gastrointestinal epithelial cells, which affect intestinal mucosal immunity and barrier integrity. They have a variety of biological activities, including improving insulin sensitivity, inducing gluconeogenesis, and weight loss [20]. Compared with the control group, the contents of acetic acid, propionic acid, butyric acid, isovaleric acid, and valeric acid in the model group were remarkedly reduced (*p* < 0.05, *p* < 0.01).Compared with the model group, the contents of acetic acid, butyric acid, valeric acid, and isovaleric acid were remarkedly increased in the ZPH and ZPM groups (500 and 250 mg/kg) (*p* < 0.05, *p* < 0.01) (Figure 7a–g). The results indicated that the hazel leaf polyphenol extract could promote the contents of SCFAs in the obese mice gut.

### 2.8. Effects of ZP on White Adipose Tissue Protein Expression

**Figure 8:**
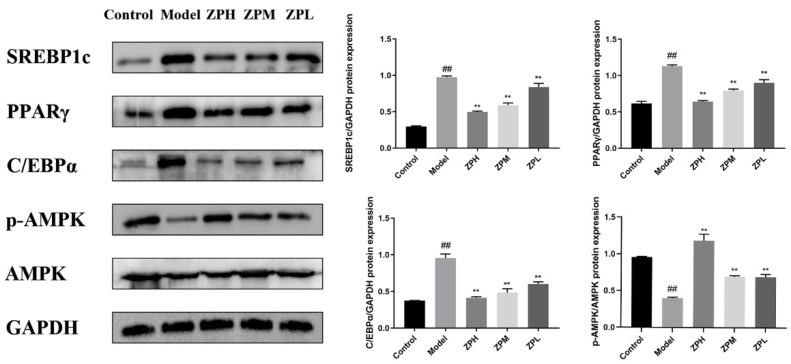
Effects of ZP on white adipose tissue protein expression. ## *p* < 0.01 compared to control group. ** *p* < 0.01 compared to model group.

As shown in Figure 8, the expression levels of lipid synthesis-related proteins SREBP1c (sterol-regulatory element binding protein 1c), PPARγ (peroxisome proliferator-activated receptor gamma), and C/EBPα (CCAAT enhancer binding protein α)in adipose tissue of the model group were significantly higher than those of the control group (*p* < 0.01), while the protein expression levels treated with different ZP concentrations (500, 250, and 125 mg/kg) were significantly lower than those of the model group (*p* < 0.01). AMPK (adenosine 5′-monophosphate AMP-activated protein kinase) is a key target for energy regulation, and the levels of AMPK and its phosphorylated proteins were also detected. The expression of p-AMPK protein in white adipose tissue of ZP treatment groups (500, 250, and 125 mg/kg) was significantly higher than that of the model group (*p* < 0.01), which was close to that of the control group (*p* < 0.01). This suggested that the anti-obesity effect of hazel leaf polyphenol extract might be associated with the inhibition of the expression of lipid synthesis protein and AMPK phosphorylation.

### 2.9. Effect of ZP on Lipid Levels in 3T3-L1 Cells

**Figure 9:**
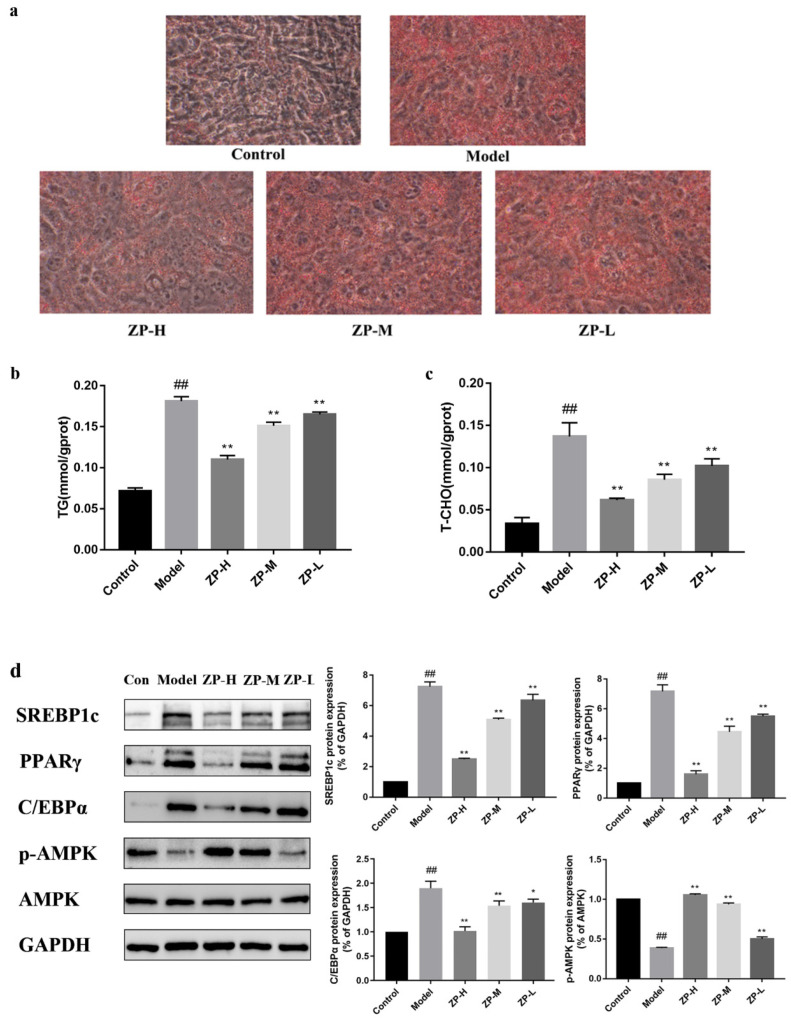
Effects of ZP on 3T3-L1 cells. (**a**) Cell oil red O staining (400×). (**b**) TG. (**c**) T-CHO. (**d**) Western blot. ## *p* < 0.01 compared to control group. * *p* < 0.05, ** *p* < 0.01 compared to model group.

The effects of different concentrations of ZP on 3T3-L1 cell viability were measured by MTT (Figure S2), and subsequent cultures were performed at concentrations of 50, 30, and 10 μg/mL. As shown in Figure 9a, the number of lipid droplets decreased with the increase in the administered dose after the treatment of ZP. This indicated that hazel leaf polyphenol extract could slow down the formation of lipid droplets. Compared with the control group, the content levels of TG and T-CHO in the cells were increased obviously (*p* < 0.01), while the content levels of TG and T-CHO in the cells treated with ZP were obviously decreased compared with the model group (*p* < 0.01) (Figure 9b,c). This suggested that hazel leaf polyphenol extract could inhibit the synthesis of lipids in cells.

Therefore, the expression levels of SREBP1c, PPARγ, and C/EBPα proteins related to lipid synthesis were further detected in 3T3-L1 cells. Compared with the control group, the protein expression levels of SREBP1c, PPARγ, and C/EBPα in the cells were significantly higher in the model group (*p* < 0.01). Compared with the model group, the protein expression levels treated with different ZP concentrations (500, 250, and 125 mg/kg) were significantly reduced (*p* < 0.01) (Figure 9d). In addition, the expression level of p-AMPK in the model group was significantly lower than that in the control group, and the phosphorylation level of AMPK was significantly increased after ZP treatment. These results suggested that hazel leaf polyphenol extract could inhibit the expression of lipogenic genes and promote the activation of AMPK in 3T3-L1 cells. This is consistent with in vivo results.

### 2.10. Molecular Docking Analysis

**Figure 10:**
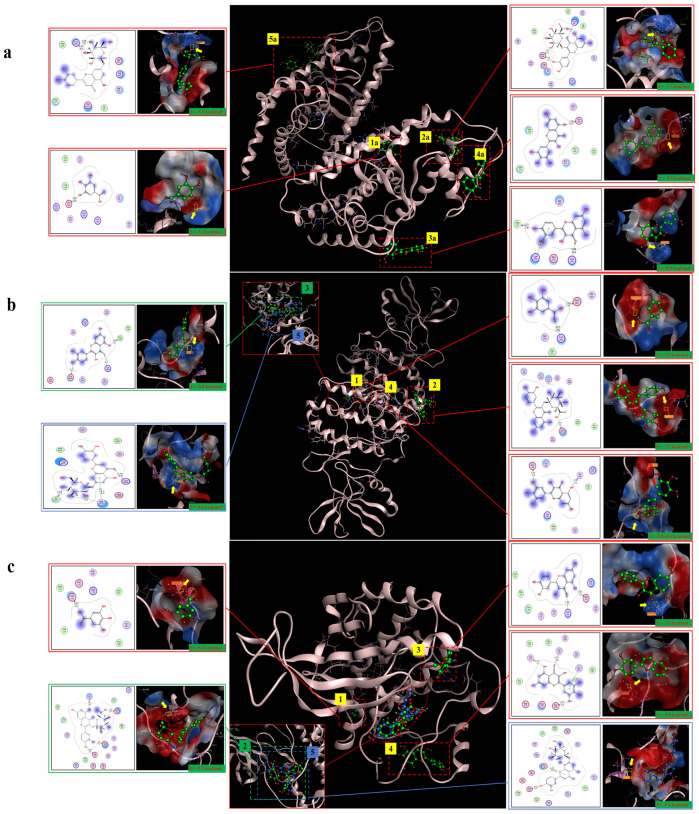
The five compounds and target binding conformation.(**a**) PPARγ. (**b**) C/EBPα. (**c**) AMPK. Label 1 represents gallic acid, label 2 represents quercetin-3-O-beta-D-glucopyranoside, label 3 represents quercetin, label 4 represents myricetin, and label 5 represents luteolin-7-O-glucoside.

The molecular docking results showed that gallic acid, quercetin-3-O-beta-D-glucopyranoside, quercetin, myricetin, and luteolin-7-O-glucosidein had good binding with PPARγ, C/EBPα, and AMPK, respectively, according to the binding energy less than or equal to −4 kcal/mol. Their binding sites and binding forms are shown in Table 1. Each of the above compounds binded well to PPARγ, C/EBPα, and AMPK, and the specific binding conditions are shown in Figure 10. These results indicated that gallic acid, quercetin-3-O-beta-D-glucopyranoside, quercetin, myricetin, and luteolin-7-O-glucosidein in hazel leaf polyphenol extract might be the main active components affecting lipid metabolism and anti-obesity.

## 3. Discussion and Limitation

### 3.1. Discussion

Obesity is becoming one of the most important metabolic diseases worldwide. The prevalence of overweight or obesity is increasing in both developed and developing countries [21]. The adverse effects of diet pills have prompted the search for safer natural medicines. Natural polyphenols are a class of compounds widely found in plants with antioxidant, anti-inflammatory, and antibacterial properties. In a previous study, we extracted and determined the polyphenols in hazel leaves, characterized their content, and verified their good antioxidant and anti-inflammatory activities. In addition, we predicted their potential therapeutic effects on dyslipidemia, atherosclerosis, and diabetic complications using targeted network pharmacology [15]. Therefore, in the present study, the anti-obesity effects of hazel leaf polyphenol extract were preliminarily investigated using animal and cellular experiments combined with molecular-docking techniques based on network pharmacology.

Based on the results of network pharmacological analysis, the core targets of PPARγ and the KEGG-enriched pathway of PPAR signaling pathway attracted our attention and were further validated by in vivo and in vitro experiments. Adipose tissue in obese mice expands by increasing the size and number of adipocytes. When energy intake exceeds energy expenditure, fat is stored in the adipose tissue, leading to hypertrophy and weight gain [22]. In this study, the body weight and adiposity of mice in the model group were significantly increased after a high-fat diet. HE staining of adipose tissue also showed that the cell volume and number were significantly larger in the model group than those in the control group. The hazel leaf polyphenol extract intervention improved the above indices in obese mice, suggesting that they could inhibit weight gain by reducing the volume and number of adipocytes. Obesity is often associated with dyslipidemia, and elevated TC and TG can promote atherosclerosis [23]. Serum biochemical results showed that high-fat diets resulted in dyslipidemia (TG, T-CHO, LDL-C, and HDL-C) and abnormal liver function (ALT and AST) in mice. Further HE and oil red O-stained pathological sections also confirmed the formation of lipid accumulation in the liver and adipocytes of mice in the model group. Serum lipid levels, as well as lipid accumulation in the liver and adipocytes, were improved in obese mice treated with hazel leaf polyphenol extract, suggesting that hazel leaf polyphenol extract has anti-obesity ability.

Studies have shown that the gut flora is inextricably linked to obesity and related metabolic diseases [24,25]. Gut flora, the microbiota residing in the host’s large and small intestines, is essential for maintaining intestinal immune homeostasis and nutrient absorption and also participates in processes related to body energy metabolism by regulating the body’s metabolic axis. Clinical data show that the diversity of gut flora is significantly lower in obese individuals than in normal-weight individuals [26]. We examined the intestinal flora of mice and found that supplementation with hazel leaf polyphenol extract ameliorated the changes in the diversity and structure of intestinal microorganisms induced by a high-fat diet, and they reduced the ratio of Firmicutes/Bacillus phylum, an important marker of intestinal homeostasis. In turn, a lower F/B value implies a reduction in fat accumulation, as well as an improvement in lipid metabolism and hepatic steatosis [27,28]. At the genus level, hazel leaf polyphenol extract intervention reduced the abundance of *Oscillibacter* and *Desulfovibrionaceae* and enriched Lactobacillus, *Alloprevotella,* and *Muribaculaceae*. Previous studies have shown that *Alloprevotella* and *Muribaculaceae* are negatively correlated with metabolism-related indices (i.e., body weight, fat mass, and glucose metabolism). *Oscillibacter* is negatively correlated with some indices of intestinal barrier and mucus function [29]. These suggested that hazel leaf polyphenol extract might modulate the gut flora to exert anti-obesity effects.

Gut microbial fermentation of dietary fiber produces short-chain fatty acids, whereas a high-fat, high-protein diet leads to a reduction in short-chain fatty acid production [30]. Further testing of short-chain fatty acid content was found to be consistent with a reduction in short-chain fatty acids in the feces of the obese mouse model group induced by the high-fat diet in this study. Alterations in intestinal flora affect changes in their products’ short-chain fatty acids. *Lactobacillus* promotes the production of short-chain fatty acids and maintains the stability of the internal intestinal environment, which is thought to attenuate high-fat diet-induced obesity and related complications [31]. Short-chain fatty acids offer potential therapeutic targets for the treatment of obesity and are related to metabolic diseases by altering host metabolic pathways [32]. Among them, butyric acid affects obesity by modulating the function of the gut barrier-activated peroxisome proliferator γ (PPARγ) [33]. The intervention of hazel leaf polyphenol extract increased the content of various short-chain fatty acids, which may be one of the ways to improve obesity. Meanwhile, short-chain fatty acids directly bind and activate extracellular G protein-coupled receptors [34] and mediate phosphorylation of the AMPK pathway [35]. It is a substrate for energy metabolism in the tricarboxylic acid cycle and regulates protein kinase (AMPK) activation via AMP to maintain homeostasis in the body environment [36]. Short-chain fatty acids promote oxidation and browning of 3T3-L1 adipocytes and inhibit lipid accumulation via the β3-adrenergic receptor/AMP-activated protein kinase α signaling pathway [37].

The PPAR signaling pathway is widely regarded as a potential mechanism for the hypolipidemic effects of Chinese herbal medicines. PPARγ is mainly found in adipose tissue and is an important mediator of energy homeostasis and cell differentiation. It promotes adipose differentiation and lipid synthesis, leading to morphological changes and adipocyte enlargement, and is also an important target for the treatment of type 2 diabetes mellitus [38]. PPARγ is a key transcription factor for adipocyte differentiation, activating the expression of a series of adipocyte phenotypic genes, thus regulating the whole process of preadipocyte to adipocyte differentiation. Western blot analysis of mouse adipose tissue revealed a significant increase in PPARγ protein expression in the model group, which was altered by the administration of hazel leaf polyphenol extract. In general, a high-fat, high-calorie diet modulates the expression of certain lipoproteins, leading to fat accumulation [39]. SREBP-1 is the major transcription factor for lipogenic gene expression in white adipose tissue. Its major isoform, SREBP-1c, is expressed in the liver and white adipocytes and promotes adipogenesis through the activation of triglyceride synthesis genes [40]. C/EBPα is also a key regulator of preadipocyte to mature adipocyte differentiation and is directly involved in cell differentiation by regulating the expression of genes related to intracellular glucose metabolism [41]. Meanwhile, C/EBPα is expressed prior to the initiation of transcription of most adipose-specific genes and is an important regulatory transcription factor for pro-adipocyte differentiation and lipid synthesis [42]. Our results also showed that ZP inhibited the overexpression of SREBP1c and C/EBPα proteins in adipocytes of mice on a high-fat diet, in agreement with previous studies [43], which may be one of the reasons for the reduction in lipid deposition by hazel leaf polyphenol extract.

AMPK is an important target of energy regulation and one of the key pathways in the regulation of lipid metabolism. AMP-dependent protein kinase phosphorylation reduces the incidence of obesity through the down-regulation of its downstream adipose generation-related genes, such as SREBP-1c and C/EBPα, to reduce the incidence of obesity [43,44]. We further examined the protein expression of AMPK and found that hazel leaf polyphenol extract was able to regulate AMPK phosphorylation levels.

In addition, we replicated the lipid differentiation process of 3T3-L1 cells. It was found that the cells were rounded and produced distinct lipid droplets upon maturation, while the administration of hazel leaf polyphenol extract significantly reduced the accumulation of lipid droplets. Meanwhile, the expression of PPARγ, SREBP-1c, C/EBPα, and AMPK proteins in the cells was consistent with the in vivo results, suggesting that hazel leaf polyphenol extract inhibited lipogenesis, which was similar to the results of other studies [45]. Moreover, five compounds of gallic acid, quercetin-3-O-β-D-glucoside, quercetin, myricetin, and lutetin-7-o-glucoside in hazel leaf polyphenol extract were simulated and screened by using the molecular docking technique and were well bound to PPARγ, C/EBP-α, and AMPK. They may be the key effector components.

### 3.2. Limitation

No anti-obesity experiments so far have been conducted for these components either individually or in combination, and no comparison has been made with the effect of hazel leaf polyphenols extract, which must be validated to support the conclusion of this study.

## 4. Materials and Methods

### 4.1. Materials and Reagents

Referring to our previous study [15], after the freeze-dried hazel leaves were crushed, 1.0 g of hazel leaf powder was added to 15 mL of a mixture of 1% hydrochloric acid and 70% methanol (1:1, *v*/*v*), vortexed for 30 s, sonicated for 30 min, and then centrifuged at 1800× *g* for 10 min. The extraction was repeated twice. The lyophilized supernatant and residue were extracted under different acid–base conditions to obtain the hazel leaf polyphenol extract (ZP, 10.5%) and stored at −80°C. The yield of ZP was evaluated by calculating the weight ratio of freeze-dried extracted ZP and leaf powder. The yield of ZP was 10.5%. The ZP was analyzed via chromatographic fingerprint using the UPLC-TOF-MS/MS system (Waters Q-TOF SynaptG2 high-resolution mass spectrometer and Waters ACQUITY UPLC system, Water, Milford, MA, USA) with chromCore 120 C18 column (1.8 µm, 2.1 × 100 mm), equipped with electrospray ionization (ESI). With the automatic matching function and the database UNIFI 1.6 software (Waters, Milford, MA, USA), compounds can be identified quickly. The parameters are set as follows: the analysis time range is 1–25 min. The allowable mass error range is ±10 ppm, the quality detection range is 50–1500 Da, and the negative adduct contains H- and HCOO-. The combined peak area of high-resolution mass spectrometry is used for semi-quantitative analysis [15]. Based on previous results, seventeen compounds were obtained from the identification, and their relative contents are shown in Table S1. Their original chromatograms are shown in Figure S1 [15].

Triglycerides (TGs), total cholesterol (T-CHO), high-density lipoprotein cholesterol (HDL-C), and low-density lipoprotein cholesterol (LDL-C) were obtained from Nanjing Jiancheng Institute of Biological Engineering (Nanjing, China). Insulin, 3-(4,5-Dimethylthiazol-2-yl)-2,5-diphenyltetrazolium bromide (MTT), BCA protein assay reagent kits, and penicillin/streptomycin were purchased from Beijing Solarbio Science & Technology Co., Ltd. (Beijing, China). 3-Isobutyl-1-methyl-2,6 (1H,3H)-purinedione (IBMX) and dexamethasone were obtained from Sigma Aldrich Inc. (St Louis, MO, USA). Orlistat was purchased from Hunan Mingrui Pharmaceutical Co., Ltd. (Liuyang, China).

### 4.2. Network Pharmacology Analysis

The chemical formulae of seventeen compounds in the ZP obtained by UPLC-TOF-MS/MS analysis were entered into Pubchem [46] (https://pubchem.ncbi.nlm.nih.gov/, accessed on 22 December 2023), and the two-dimensional structural diagrams of the chemical components were downloaded and imported into the Swiss ADME [47] (http://www.swissadme.ch/, accessed on 23 December 2023) system for activity screening (screening criteria: GI absorption, high; "Yes" in the Drug likeness box with a number greater than or equal to one-half of the items). The obtained active ingredients were entered into the Swiss Target Prediction Database [48] (http://swisstargetprediction.ch/, accessed on 23 December 2023) for target prediction, and the targets with a probability ≥ 0 were selected to obtain the gene names of the component targets. The keyword "obesity" was also used for disease target mining in GeneCards database (https://www.genecards.org/, Revelance Score ≥ 0.104990125), OMIM database (https://www.omim.org/, accessed on 25 December 2023), and DisGeNET database (https://www.disgenet.org/, Score ≥ 0.08). The disease targets were compared with the phenolic targets in Venny 2.1.0 (https://bioinfogp.cnb.csic.es/tools/venny/, accessed on 26 December 2023), and it was found that the cross-targets may be the key targets of hazel leaf phenolic compounds against obesity. The key targets were entered into the STRING (https://cn.string-db.org/, accessed on 26 December 2023) online website, and a protein–protein interaction (PPI) network was constructed (human source was selected; confidence level ≥ 0.9). The results were imported into Cytoscape 3.9.0 for visualization and analysis, and the core targets were filtered according to the values of "Closeness", "Betweenness", and "Degree" using the plug-in Centiscape 2.2 [49]. GO and KEGG pathway enrichment analyses were also performed using Metscape Data Platform (https://metascape.org/, accessed on 27 December 2023). Finally, GO enrichment histograms and KEGG enrichment bubble maps were plotted using Microbiology Online Platform [50] (https://www.bioinformatics.com.cn/, accessed on 28 December 2023).

### 4.3. Animals and the Experimental Design

The maleC57BL/6J mice, weighing 14–16 g, were provided by Liaoning Changsheng Biotechnology Co., Ltd. (Shenyang, China), and the certificate number was SCX (Liao) 2020-0001.This animal study was approved by the Laboratory Animal Ethics Committee of Changchun University of Chinese Medicine (Approval number: YX-2022611) and was carried out under its supervision.

All mice were fed in a constant temperature environment of 23 ± 2 °C and humidity of 55 ± 10% during the entire experiment, with light/dark cycles for 12 h and free drinking water. After one week of acclimation to the environment, the mice were randomly divided into six groups, a control group (*n* = 8), model group (*n* = 8), positive control group (Orlistat, 30 mg/kg, *n* = 8), hazel leaf polyphenol extract high-dose group (ZPH, 500 mg/kg, *n* = 8), hazel leaf polyphenol extract medium-dose group (ZPM, 250 mg/kg, *n* = 8), and hazel leaf polyphenol extract low-dose group (ZPL, 125 mg/kg, *n* = 8) based on our previous studies. All groups were fed a high-fat diet (D12492, Research Diets, New Brunswick, NJ, USA), except the control group, which was fed a regular maintenance diet. After six weeks, each group was given the corresponding dose of the subject via intragastric administration at the same time every day. The control group was given equal distilled water. The body weight and food intake of the mice were recorded every week.

After 6 weeks of administration, the mice were anesthetized with 1% pentobarbital sodium. The fat content of mice was scanned using the UltraFocus high-resolution small animal X-ray imaging system (Faxitron, Marlborough, MA, USA). Then, blood samples were taken, and the mice were euthanized. Liver tissue, perirenal white adipose tissue, and fecal samples were collected. The livers of mice were weighed, and the liver index was calculated.

### 4.4. Serum Biochemical Analysis

The blood sample was centrifuged at 3000 rpm for 15 min, and the supernatant was collected. TG (triglyceride), T-CHO (total Cholesterol), HDL-C (HDL-C, high-density lipoprotein cholesterol), LDL-C (low-density lipoprotein cholesterol), AST (aspartate aminotransferase), and ALT (alanine aminotransferase) were detected by the commercial kits according to the instructions.

### 4.5. Histological Analysis

The liver and perirenal white adipose tissue of mice were fixed in 4% (*v*/*v*) paraformaldehyde for 24 h.Then, the paraffin-embedded sections were stained with hematoxylin–eosin, or frozen sections were stained with oil red O. The tissue morphology was observed under a microscope (Nikon, Tokyo, Japan).

### 4.6. Gut Microbial Analysis

The total DNA was extracted from the resulting stool sample, and the gene was sequenced using the method of designing primers in the conserved region and adding them to the sequencing street. The results were then amplified by PCR, purified, quantified, and sequenced using the Illumina HiSeq 2500 platform. The ACE index and Shannon index were analyzed to explore the diversity of α among samples. Principal component analysis (PCA) was performed between the samples. The gut microbial of each group was analyzed at the phylum level and genus level, and the changes of the gut microbial of obese mice were screened using LEfSe analysis.

### 4.7. Determination of Short-Chain Fatty Acids (SCFAs)

The 50 mg fecal sample was placed into a 2 mL EP tube, in which 1 mL of pure water was added and vortexed for 10 s. Then, steel balls were added to the EP tube, which was treated with a 40 Hz grinder for 4 min, followed by ultrasonic treatment (ice water bath) for 5 min, repeated three times, and then centrifuged at 4 °C and at 5000 rpm for 20 min. After centrifugation, 0.8 mL of supernatant was added into another 2 mL EP tube, along with 0.1 mL of 50% H_2_SO_4_ and 0.8 mL of extract (containing internal standard 2-methylvaleric acid, 25 mg/L, methyl tert-butyl ether). The EP tube was vortexed for 10 s, oscillated for 10 min, sonicated for 10 min (ice water bath), and centrifuged at 10,000 rpm for 15 min at 4 °C.

After standing at −20°C for 30 min, the supernatant was removed from the injection bottle for GC-MS detection. The Shimadzu GC2030-QP2020 NX gas chromatography–mass spectrometer was equipped with Agilent HP-FFAP capillaries (30 m × 250 μm × 0.25 μm). The chromatographic conditions: injection volume of 1 μL, split ratio of 5:1, solvent delay time of 3.5 min. The mass spectrometry conditions: helium as the carrier gas, column flow rate of 1 mL/min, advance port temperature of 240 °C, transmission line temperature of 240 °C, quadrupole temperature of 150 °C, ion source temperature of 200 °C, and ionization voltage of −70 EV. Data analysis and chromatogram are shown in Table S5 and Figure S3.

### 4.8. Cell Culture and Treat

Mice embryonic fibroblasts (3T3-L1) were purchased from Wuhan Prosa Life Science Co., Ltd. and were cultured in DMEM culture medium (Hyclone, Logan, UT, USA) containing 10% bovine serum and 1% penicillin/streptomycin, under humidified conditions of 37 °C and 5% CO_2_ concentration. The ZP cytotoxicity was detected by MTT method. The cells were cultured in 96-well plates at 5000 per well for 24 h with ZP added and cultured for 24 h or 48 h. A total of 20 μL MTT (5 mg/mL) was added to each well, incubated for 4 h, and the medium was carefully aspirated. Then, 150 μL of dimethyl sulfoxide solution (DMSO) was added to each well and oscillated for 10 min to read the absorbance at 490 nm.

The cells were rolled into six-well plates to induce differentiation. When the cells grew to contact inhibition, differentiation was initiated (0 days). They were then cultured in a fully differentiated medium containing 0.5 mM of 3-isobutyl-1-methylxanthine, 250 nM of dexamethasone, and 10 μg/mL of insulin for 48 h (2 days), continued in a complete culture medium of 10 μg/mL of insulin for 48 h (4 days), and continued with complete culture for 48 h (6 days) [35]. The complete culture medium with or without 10 μg/mL of insulin was replaced every 2 days. Different ZP concentrations were treated on days 0–4, and the cells cultured for six days were collected for relevant index detection. The control group for cell experiments was undifferentiated 3T3-L1 cells.

### 4.9. Cell Oil Red O Staining

The cells were spread into six-well plates, induced to differentiate for about 8 days, and stained with oil red. The oil red O staining working solution was prepared with the oil red O solution and the oil red O dilution at the ratio of 3:2, mixed evenly, left for 10 min, and filtered by 0.45 μm needle for 2 h. The cell culture medium was slowly pumped, washed with PBS, fixed with 4% paraformaldehyde fixing solution for 10 min, rinsed with PBS, and coated with appropriate amount of dyeing washing solution. Then, the staining wash solution was then removed, washed with PBS for 20 s, observed under a microscope, and photographed.

### 4.10. Western Blotting

The white adipose tissue or cells were lysed in a lysate containing protease inhibitors and phosphatase inhibitors. After protein quantification, loading buffer was added, vortexed, and mixed. The parafilm was sealed and boiled in a water bath at 100 °C for 5 min to denature the protein. The proteins were isolated by SDS-PAGE and transferred to PVDF membrane, which was incubated overnight at 4 °C with primary antibodies against C/EBPα, PPARγ, SREBP1 c, AMPK, GAPDH (Proteintech Group, Inc., Wuhan, China), and p-AMPK (Cell Signaling Technology, Danvers, MA, USA).The protein expression was analyzed by binding to secondary antibodies using the BeyoECL Plus Enhanced Chemiluminescence Kit. ImageJ 1.8.0 software was used for relative expression analysis.

### 4.11. Molecular Docking Method

Molecular docking was carried out to evaluate the binding affinity between the compounds and their protein targets. All identified components of hazel leaf polyphenol extract (Table S1) were used as target components for molecular docking with proteins such as C/EBPα, PPARγ, and AMPK (no protein conformation of SREBP1c was found) to simulate the binding state at the molecular level. The structure files of the five molecules (downloaded from Pubchem: https://pubchem.ncbi.nlm.nih.gov/, accessed on 22 December 2023) were imported into MOE 2022.02 software. The ligand molecules were preprocessed using the automatic function of the software "Compute→Molecule→Energy Minimize", energy minimization, optimization, and saved as a ligand library. At the same time, the receptor proteins to be docked (downloaded from RCSB PDB: https://www.rcsb.org/, accessed on 25 December 2023) were imported into the MOE 2022.02 software. The structure of the receptor molecules was protonated using the function "QuickPrep"; the obese key target molecule (receptor macromolecule) was taken as a rigid entity, and the conformation of the active ingredient (ligand macromolecule) was changed within a certain range using the semi-flexible docking method to dock 10 different conformations. The one with a binding energy lower than "−4 kcal/mol" was selected. The binding energies, binding sites, bond lengths, and other data were recorded [51].

### 4.12. Statistical Analysis

Data were expressed as mean ± standard deviation (SD). The differences between groups were analyzed by one-way ANOVA, Tukey’s multi-comparison test, and two-way ANOVA. All data were analyzed using GraphPad Prism 8.0. A *p*-value of less than 0.05 was considered statistically significant.

## 5. Conclusions

In conclusion, this study employed network pharmacology, in vivo and in vitro assays, gut microbiota, and molecular docking to investigate the anti-obesity efficacy and possible molecular mechanisms of hazel leaf polyphenol extract. Hazel leaf polyphenol extract plays an anti-obesity role by regulating intestinal flora, short-chain fatty acids, and lipid metabolism. PPAR signaling is one of the potential anti-obesity mechanisms. Gallic acid, quercetin-3-O-β-D-glucopyranoside, quercetin, myricetin, and lutein-7-o-glucoside in the hazel leaf polyphenol extract may be the key active ingredients. However, these conclusions are based on bioinformatics databases and computer analysis and need to be further verified by experimental and clinical pharmacological data.

## Figures and Tables

**Table 1:** The binding energy of compounds and targets (kcal/mol).

Target	PDB ID	Target Structure	Compound	Affinity (kcal/mol)	Interaction	Receptor	Distance
PPARγ	2I4J	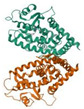	Gallic acid	−5.2	H-donor	Glu 324-OE2	2.81
			Quercetin-3-O-beta-D-glucopyranoside	−7.7	H-acceptor	Lys 457-HZ2	2.82
			Quercetin	−8.8	H-acceptor	Lys 244-HZ2	2.88
			Myricetin	−5.9	H-donor	Glu 272-OE1	2.86
			Luteolin-7-O-glucoside	−5.9	H-acceptor	Lys 263-HZ1	2.98
C/EBPα	6DC0	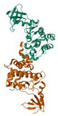	Gallic acid	−7.0	H-donor	Asp 286-OD2	2.91
			Quercetin-3-O-beta-D-glucopyranoside	−7.5	H-donor	Asp B517-OD1	2.87
			Quercetin	−6.8	H-acceptor	Lys A295-HZ1	3.00
			Myricetin	−5.8	H-donor	Asp 286-OD2	2.91
				−4.4	H-donor	Tyr 282-O	2.76
			Gallic acid	−8.6	H-acceptor	Lys A295-HZ1	4.10
AMPK	3AQV	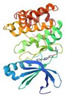	Gallic acid	−8.7	H-donor	Glu 113-OE1	2.87
			Quercetin-3-O-beta-D-glucopyranoside	−9.1	H-donor	Glu 143-OE2	2.72
			Quercetin	−7.0	H-acceptor	Lys 141-HZ3	2.99
			Myricetin	−5.8	H-donor	Asp 216-OD2	2.77
			Luteolin-7-O-glucoside	−5.4	H-donor	Asp 103-OD2	2.91

## Data Availability

Data is contained within the article or Supplementary Materials.

## Supplementary Materials

The following supporting information can be downloaded at: https://www.mdpi.com/article/10.3390/ph17101349/s1, Figure S1: The relative content of the main components in hazel leaf polyphenols; Figure S2: Evaluation of 3T3-L1 viability using MTT assay after exposure to different concentrations of hazel leaf polyphenols for 24 h or 48 h; Figure S3: Total ion flow chromatograms (TIC) of different groups of short-chain fatty acids. (a) Control group. (b) Model group. (c) ZPH group. (d)ZPM group. (e) ZPL group. Table S1: Identification of components in hazel leaf polyphenols; Table S2: The component targets; Table S3: The disease targets; Table S4: The key targets; Table S5: Quality control (RSD) and standard curves for quantitative analysis of short-chain fatty acids.

## Abbreviations

ZP, hazel leaf polyphenols; TG, triglyceride; T-CHO, total cholesterol; HDL-C, high-density lipoprotein cholesterol; LDL-C, low-density lipoprotein cholesterol; IBMX, 3-Isobutyl-1-methylxanthine; MTT, 3-(4,5-Dimethylthiazolyl-2)-2,5-diphenyl tetrazolium bromide; DMSO, dimethyl sulfoxide solution; HE, hematoxylin eosin; ALT, alanine aminotransferase; AST, aspartate aminotransferase; PCA, principal component analysis; SCFAs, short-chain fatty acids; AMPK, adenosine 5′-monophosphate (AMP)-activated protein kinase; p-AMPK, phosphorylated AMP-activated protein kinase; PPARγ, peroxisome proliferator-activated receptor gamma; C/EBPα, CCAAT enhancer binding protein α; SREBP1c, sterol-regulatory element binding protein 1c.
