# Supplementary material for: Potential Anti-Obesity Effect of Hazel Leaf Extract in Mice and Network Pharmacology of Selected Polyphenols
Source: Pharmaceuticals (Basel). 2024 Oct 9;17(10):1349. doi: 10.3390/ph17101349 (PMC11510286; doi:10.3390/ph17101349)
Supplement: Supplementary file 1 [file pharmaceuticals-17-01349-s001.zip › pharmaceuticals-3174020-supplementary.pdf]

Supplementary Material

Table S1.Identification of components in hazel leaf polyphenols

Molecule	Formula	Name	RT (min)	Major fragment ions (m/z)	contents (mg/100g of dry extract)
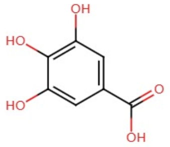	$C_7H_6O_5$	Gallic acid	1.00	125.0236	2.3
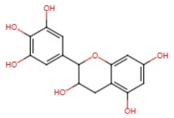	$C_{15}H_{14}O_7$	Gallocatechin	1.37	109.0220, 137.0241, 179.0306, 219.0535, 261.0735	1.2
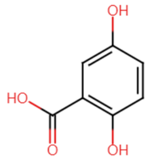	$C_7H_6O_4$	2,5-dihydroxybenzoic acid	1.50	109.0266	1.8
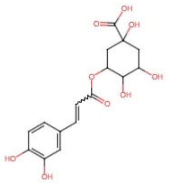	$C_{16}H_{18}O_9$	Chlorogenic acid	1.68	191.0518, 192.0529, 161.0183	1.3
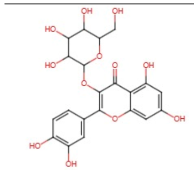	$C_{21}H_{20}O_{12}$	Quercetin-3-O-beta-D-gluc opyranoside	2.35	300.9928	3.2

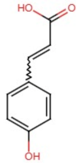	$C_9H_8O_3$	p-Coumaric acid	2.43	119.0476	2.3
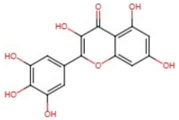	$C_{15}H_{10}O_8$	Myricetin	2.61, 6.38	165.0543, 179.0658, 288.1270, 315.0105	5.2
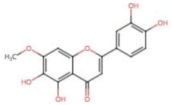	$C_{16}H_{12}O_7$	Pedalitin	3.11, 8.8	271.0560, 301.0004, 311.0933	1.1
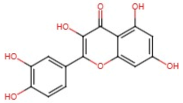	$C_{15}H_{10}O_7$	Quercetin	4.97, 19	149.0549, 178.9954	6.4
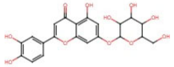	$C_{21}H_{20}O_{11}$	Luteolin-7-O-glucoside	9.20	285.1414, 327.1232	5.8
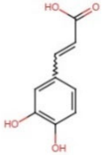	$C_9H_8O_4$	Caffeic acid	2.30	135.0453	2.1

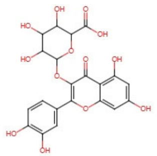	$C_{21}H_{18}O_{13}$	Quercetin-3-O-beta-D-gluc uronide	2.61	121.0314, 135.0453, 163.0385, 201.0195, 301.0004	2.6
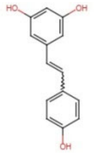	$C_{14}H_{12}O_3$	Resveratrol	2.92	120.0543, 153.0203, 165.0543, 183.0832, 225.0572	1.5
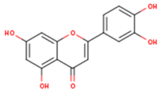	$C_{15}H_{10}O_6$	Luteolin	5.86	241.0591	2.6
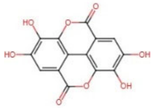	$C_{14}H_6O_8$	Ellagic acid	8.72	185.9775, 257.0444, 299.1262	2.2
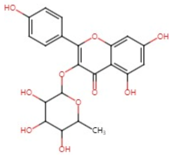	$C_{21}H_{20}O_{10}$	Kaempferol-3-O-rhamnosid e	4.74, 7.41	227.0676, 255.0311, 285.0396	5.7
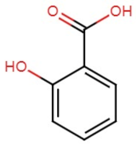	$C_7H_6O_3$	Hydroxybenzoic acid	1.94	79.0213, 93.0343, 109.0311	1.9

Table S2.The component targets

Components targets (539)									
KDM4E	LGALS9	TLR4	LGALS8	CDC7	PLK4	FAAH	CES2	SCD	PTGES
TERT	CHEK1	FFAR1	IKBKB	AURKA	KDM6B	HDAC3	PLA2G10	PDPK1	CA12
SLC37A4	AKR1B10	LDHA	DHFR	GSR	ABL1	EGFR	F2	C1R	F10
PLG	CA1	CA2	NRAS	HRAS	TNF	TTR	ALB	ESR1	MMP1
HMGCR	RAF1	PLA2G1B	GBA	FUCA1	NR3C1	SHBG	ERBB2	AMY1A	AMY1B
AMY1C	CYP1A1	TYMS	ATP1A1	APP	ARG1	ALDH2	CYP17A1	SERPINE1	PRKCG
MPO	CYP1A2	ALPL	FGF1	JUN	FABP3	ITGB1	ITGA4	PRKCB	INSR
LCK	FYN	BCHE	PGR	CDK1	PYGL	POLB	ITGAV	ITGB3	KLK1
EPHX1	FABP1	LDHB	ADH1A	CAPN1	CA3	CTSL	CTSB	HSP90AA1	LYN
IGF1R	CHRM2	CHRM4	ABCB1	SERPINA6	NR3C2	HSP90AB1	ELANE	MMP2	MMP3
CTSG	ITGA2B	PTPRC	MET	CYP3A4	F7	STS	CHRM5	ADRA2A	FGF2
MMP7	FBP1	HMOX1	PDGFRB	CTSH	FGR	PARP1	POLA1	ALOX5	ADORA3
TMIGD3	HSPA1A	AR	BCL2	PTPRF	CYP2D6	MAPT	KIT	HSPA5	HSPA8
CHRM1	PIM1	FGFR1	TOP1	TOP2A	G6PD	VDR	ESRRA	CYP2A6	CYP19A1
CYP2C9	CDK4	ODC1	IMPDH2	SRC	CFTR	F3	SLC5A1	HSD17B1	SELL
MIF	DRD2	AKR1A1	PLA2G2A	HSP90B1	TYR	MMP9	GABRA1	GABRB2	GABRG2
DAO	BRAF	FABP4	AKR1B1	CYP11B1	VEGFA	ST6GAL1	ALOX15	NQO2	SELP
CBR1	MGMT	SELE	UGT2B7	PRKCA	AKR1C4	CAPN2	PTPN2	LGALS3	IGFBP3
FLT1	PTPN1	ALOX12	ITGB5	ADRA2B	SRD5A1	GABRB3	GABRA6	ADRA2C	CYP11B2
RXRA	KLK2	CD22	CHRM3	IMPDH1	MAOA	TACR2	CNR1	DRD1	TBXA2R
DRD4	DRD5	COMT	KCNA3	FUT4	GART	ACHE	KCNA5	CA4	MMP8
CES1	PTGS1	CA6	GLRA1	GLRA2	RPS6KB1	JAK1	SLC6A2	CCND1	TBXAS1
ACP1	PRKCH	CCNC	CDK8	CCNE1	CDK2	CDK3	CCNA1	CCNA2	CXCR1

CXCR2	ADRA1D	EDNRA	PTAFR	BRD2	CTSS	NPY1R	ITGB7	DNMT1	MAOB
MAPK3	APEX1	PIK3R1	PSMB5	HTR1B	HTR2A	HTR2C	GABRA3	MAPK1	HSD11B1
CD38	ADORA2A	ADORA2B	PTPN6	MPG	CASP1	NOS3	NOS1	TYK2	WEE1
CDC25A	CDC25B	CDC25C	BDKRB2	AVPR2	AXL	TSPO	ADORA1	ALDH3A1	SRD5A2
SLC5A2	GABRA5	SLC6A4	AKT1	ATIC	CCKBR	CCR1	CHRNA3	CHRNB4	CYP2C19
ABCC1	EPHX2	CNR2	PTGER1	CRHR1	CA5A	NOS2	ADRA1A	PTGS2	OPRM1
RORA	PTGER4	DRD3	GCK	SOAT1	PPM1A	AHR	FLT4	KDR	CHRNA7
NUDT1	FLT3	HSD17B3	HSD17B2	PPARG	FDFT1	SNCA	PLA2G5	MMP12	ADH7
STAT3	OPRD1	OPRK1	HNF4A	IARS1	MAP3K8	GRM5	HTR2B	CCR2	STAT1
AKR1C3	PIK3CA	PIK3CB	CASP3	PRCP	HTT	PTGFR	PTGER3	PTGER2	PTGIR
CA7	CTSK	GRK6	SYK	CHRNA4	CHRNB2	MMP13	MAPK8	MAPK9	PLA2G4A
GABRA2	XDH	MTNR1A	PREP	LSS	PIK3CG	TDO2	NPY2R	MTNR1B	FNTA
FNTB	CXCR3	CLK1	PGF	PSEN1	PSEN2	PSENEN	NCSTN	APH1A	APH1B
GSK3B	MMP14	HTR6	METAP2	HLCS	RORC	TPMT	CCR4	CCR8	RPS6KA3
NEK2	PGD	JAK3	AKR1C2	PLK1	DAPK1	CTSC	MAPK10	SLC16A1	ATP12A
EPHB4	NR1H2	VCP	F2RL1	BRPF1	CASP7	CASP6	ADK	LGALS4	BACE1
TAS2R31	CD81	IL2	EIF4A1	CXCR4	YWHAG	CALM1	CALM2	CALM3	TNNC1
TNNT2	TNNI3	PPP2CA	CSNK2A1	PRKDC	HSD11B2	CDK6	PDE1B	AMPD3	FABP5
SLC6A3	NAAA	DHODH	PRKCE	TEK	PPARD	RELA	FOLH1	PRKCQ	GLO1
AKR1C1	DNM1	PTK2	LIPE	PRKCD	PTPN11	BCL2L1	MCL1	PPARA	PDE4D
FUT7	ST3GAL3	KCNMA1	KCNH2	TRAP1	NR1H3	GRM1	PTPRS	ILK	TUBB3
CAMK2B	NAE1	DYRK1A	CACNA1C	PDE7A	CBFB	PTK2B	SQLE	USP10	CASP8
GRM4	NR1I3	CDK5R1	CDK5	PDK1	SHH	LTB4R	MYLK	NPY5R	NFE2L2
PKN1	MAPK14	NTRK2	MAPKAPK3	HIF1A	CYP1B1	CA9	CYP51A1	PFKFB3	SLC22A6
PDCD4	TNNI3K	LRRK2	KDM4D	NOTUM	PAOX	TRPM8	RASGRP3	SIRT2	CA13

TRPV1	TRPV3	TTL	PIP4K2C	HCAR2	GPBAR1	CCNB3	CCNB1	CCNB2	NEU4
ESR2	HDAC2	USP13	AURKB	F2RL3	NR1H4	SLC22A12	MAP3K14	P2RX7	MGLL
SIGMAR1	SLC29A1	EPAS1	SMO	HSD17B14	MKNK1	HDAC8	FTO	NMUR2	EGLN1
TNKS2	KDM4C	RNPEP	TUBB1	DCTPP1	SLC28A3	MKNK2	GPR35	NEK6	PDE7B
NOX4	GPR84	TLR9	SLC5A4	SAE1	UBA2	HDAC6	GABBR1	DBF4	NPC1L1
CA14	ALK	ABCG2	PDE10A	HPSE	CA5B	KDM2A	DYRK1B	KDM3A	

Table S3. The disease targets

Disease Targets (1136)									
MC4R	LEP	POMC	PPARG	LEPR	FTO	PCSK1	UCP3	ENPP1	GHRL
ADRB3	UCP2	INS	CAST	LOC101929710	MC3R	BDNF-AS	ADIPOQ	BDNF	SIM1
ADCY3	NR0B2	AGRP	GNAS	AFF4	KIDINS220	LOC129933280	NUDC	CARTPT	RETN
H19	PRMT7	NTRK2	ABCC8	FFAR4	SH2B1	IL6	CENPO	LOC108167315	GHRLOS
SDC3	PHIP	CDKAL1	AOMS1	CEP19	AQP7	TUB	BBIP1	DYRK1B	INPP5E
CRP	IGF1	AOMS2	PYY	TNF	MEG3	DNMT3A	MT-CYB	SLC6A14	ATP6V0D1DT
BRCA2	MTTP	SCAPER	OBHP	UCP1	SERPINE1	HSD11B1	LOC105376020	POGZ	PDSS1
DNM1L	NRXN1	GLDC	FBXO11	KCNH2	SCN1A	POLG	LEPQTL1	MYH9	DDHD2
GCG	ALMS1	NPY	EMC1	ADRB2	LINC01672	LPL	BMIQ7	BBS1	GRIA4
DIXDC1	RSRC2	POLGARF	MIR143	IRAK1BP1	MIR122	PPARGC1A	IRS1	SMAD5-AS1	CNR1
BBS2	VPS13B	MIR27B	NR3C1	BBS4	RIC3	APOB	INSR	SLC2A4	NAMPT

MKKS	LIPE	PPARA	GNB3	SNHG11	CCK	SHBG	BBS10	RBP4	MIR33A
CCL2	GHR	APOE	LIPC	TTC8	GH1	TP53	CELA2A	GCK	TCF7L2
SREBF1	BBS7	ACE	RARRES2	IGF2	BBS9	IRS2	PTPN1	IL1B	TMEM18
TRAPPC9	DLK1	PRL	APOA1	AHDC1	HNF1A	CPE	KCNJ11	MAGEL2	ALB
BBS5	PHF6	WT1	HDAC8	COMT	PNPLA3	PNLIP	HCRT	PLIN1	IGFBP1
CYP19A1	GPT	MRAP2	HNF4A	AKT1	IL18	PNPLA2	ASOBS	CAPN10	PAX6
SNRPN	BBS12	ICAM1	EIF2S3	PON1	FABP2	IGFBP3	ADIPOR1	STAT3	FGF21
NOS3	NPY2R	LEPROT	ITLN1	APOA5	FASN	PPARD	MMP9	INSIG2	GHSR
LAS1L	FNDC5	FABP4	GHRH	AGT	DPP4	DRD2	MIR125A	CTNNB1	SOD2OT1
CHKBCPT1B	ARL6	ESR1	MIR21	CETP	IL10	SOCS3	LMNA	TBC1D2B	ADIPOR2
BGLAP	PIK3CA	TGFB1	IAPP	IPW	SIRT1	FAAH	IFT172	CLOCK	CD36
GLP1R	LOC129996745	MTOR	MIR126	SDCCAG8	GGT1	CRH	SCD	CERNA3	C3
CFAP418	HTR2C	SERPINA1<br>2	MFN2	NPAP1	PRKAR1A	APOC3	CEP290	TLR4	LCN2
LINC00237	MCHR1	SLC17A5	PPY	LZTFL1	MTHFR	TMEM67	GNPDA2	IFNG	TBX3
NDN	APLN	PRDM16	TLR2	MLXIPL	MKRN3	ASIP	BRAF	FGFR1	NLRP3
MKS1	PWAR1	ZDHHC24	IGFBP2	SLC30A8	ADRB1	MIR146B	PTEN	MIR34A	DGAT1
AKT2	H6PD	EDN1	CXCL8	NFE2L2	CIDEA	IFT74	LINC02605	MAN1B1	GAL
NMB	MIR155	HNF1B	CCDC28B	NFKB1	CREBBP	GAD2	RAB23	BMIQ8	PLA2G7
AHSG	GIPR	INPPL1	OXT	ACACB	IFT27	CDKN2A	GIP	HTR2A	PPARGC1B
ABCA1	MIR27A	MIR17	PWRN1	SLC2A2	ENPP2	IGF1R	MIR29A	GCKR	NEGR1
REN	SOCS1	PDX1	MT-TP	MYT1L	ANGPTL4	VDR	NR1H2	GAS5	RXRA
NPY5R	SLC6A3	WDPCP	CCL5	KCNQ1OT1	PGR-AS1	MIR222	LPA	PRKAB1	MC2R
APOA4	MIR223	NHLH2	FLT1	SELE	AGTR1	FMR1	TNFRSF1B	SST	IRX3
ATRX	MIR140	TRH	MIR221	MIR22	HDAC4	NTS	RPGRIP1L	IL6R	PRKACA

ACP1	CPT1A	PRKAA2	HSD11B2	ACACA	SOD2	CFD	MIR148A	CYP3A4	PRKAA1
PIK3R1	NR1H4	USP8	HBEGF	CCKAR	TMX2CTNND1	CYP2E1	LCAT	SLC6A2	PAX4
MIR146A	IL1A	F2	NEUROD1	VCAM1	THBS1	SLC2A1	MIR342	AGER	MIRLET7D
GCGR	MSTN	CNTF	LOC106728418	IGF2BP2	TRIM32	IL17A	SERPINA6	CAV1	ADRA2A
STEAP4	JAK2	HIF1A	MIR142	PDE11A	SLC6A4	MIR18A	DRD4	CEBPA	TIMP1
AR	MLN	FST	HMOX1	MAOA	SCARB1	DEAF1	CBS	MIR10B	SGK1
PMCH	LINC-ROR	MTNR1B	MTCH2	MIR23A	MIR33B	IL15	GPD1	KNG1	SMARCA4
EP300	MIR214	FADS1	NR3C2	PLTP	LIPG	LPIN1	PTH	CRHR1	MIR150
FOXC2	FOXO1	AOC3	MIR7-3HG	MIRLET7C	ACE2	PTGS2	TULP1	HFE	MIR181A1
PTPN11	NPY1R	DIO2	MIR192	GSTM1	EDNRA	G6PC1	MIR106B	MIRLET7B	VEGFA
F7	CS	MAPK8	ARMC5	CLPS	HERC2	SNORD115-1	KSR2	MIR25	GAPDH
APOA2	NPPB	MIR378A	GNRH1	MEGF8	SNORD1161	CDH23	OCA2	IL4	CNR2
MMP3	RPS6KB1	GDF15	PVT1	MIR335	MALAT1	SORBS1	MIR26B	GRP	MC1R
FGFR3	NMU	CPT1B	SIM1-AS1	NPR3	KIF7	SLC32A1	CCL11	BMAL1	TNFRSF11B
APOC1	PRKCZ	RAI1	NPHP1	WFS1	TFAP2A	ACTB	SNHG14	ITGAM	GSK3B
SPP1	PDK4	PLIN2	BCHE	WAGRO	SEC16B	ERBB2	DGAT2	CYP7A1	SYNE2
SNORD15A	MCHR2	ASTN2	PER2	MIRLET7A1	CYP21A2	HK2	CCR2	SFTA3	MIR30E
IL1R1	MYD88	BRS3	NPPA	MIR30A	MIR181C	USP48	UCN	IKBKB	XIST
HP	FBN1	MIR99A	PLA2G4A	CEBPB	BCL2	PLAT	HMGCR	KCTD15	KCNQ1
FOXA2	MIR93	SIRT3	MIR532	FABP1	SLC27A1	KISS1	F2R	MECP2	CPT2
ETV5	MC5R	G6PC3	FAIM2	TFAM	LRP1	MIR23B	FOS	CAPN5	RTL1
NPR1	FDFT1	THRA	MIR483	PHF21A	NCOA3	G6PC2	TUG1	MGAM	MIR320A
XBP1	TERC	SRC	MIR210	CRHR2	MIR193A	PCSK2	SCLT1	RET	MIR151A
RHOA	MIF	FABP3	CREB1	SOD1	SELL	PCK1	IL2	KLB	UBE3A
BMIQ11	ARMS	ART	BMIQ19	BMIQ20	BMIQ4	BMIQ9	C3orf34	C6orf117	CART

DEL11p14p12	ELA2A	GDFD	MIRK	MORMS	NEC1	O3FAR1	OB	OBAIRH	OBR
PDNP1	PPARG1	RDOB	SHP	SYND3	SYNX	TRKB	AGRT	AOMS3	AOMS4
BMIQ14	BMIQ18	GPR120	JBTS1	LEPD	LEPRD	MOSPGF	NPPS	OBHD	PC1
PPARG2	SDCN	SINO	CIMT1	CORS1	DEE58	M6S1	PC3	PGR4	VENARG
PCA1	GLM1	BMIQ12	BMIQ10	ARHR2	COLED	LBP	ACSL1	FOXO3	GPX3
CD40	HADH	GLUL	TF	NEIL1	NPC1	AHR	KCNMA1	HOXB5	PACS1
DPYD	RMST	LDLR	PRKCH	TBC1D1	HSPA5	SAT1	NR1I3	CTSS	CYP1B1
STS	TRPV1	NR1I2	CD163	CD68	CES1	CTF1	OGG1	RAPGEF3	GPX1
ACHE	SLC22A1	VLDLR	ADH1B	PARP1	DDIT3	ZNF169	FCGR3B	FTL	IDO1
MIR130B	MRC1	PFKFB3	PRKAR2B	TFRC	CA3	AKR1C3	ALDH1L1	LACTB	COX7C
CPB2	APCDD1	PPM1L	CYB5A	NQO1	ECHS1	EFNB1	ESR2	ETFDH	RAB21
ZFR2	GFPT1	HMGB2	ACADM	MIR130A	MIR184	ME1	ALDH6A1	MMUT	ZFHX3
ACLY	NUCB2	ZBTB7B	DCXR	ACP5	CYCS	FGGY	CYP26B1	ATPAF1	SLC22A3
SLC22A2	UQCRC2	AKAP1	CASP1	GAS7	PEX11A	SUCLG2	SUCLA2	WNT3A	SLC16A7
ENTPD6	SIRT6	SERPINF1	STK11	GOT2	PRKCB	CST3	ANGPTL6	NR1H3	HRH3
ANKRD26	NNT	GUCY2C	PRLH	SNAP25	TSC1	ALOX12	GLRX	HK1	NOS1
PON2	PRKG1	SREBF2	XDH	CRHBP	DCN	DRD1	GABRA6	PRLHR	ATP5F1B
KDM3A	PROX1	BAD	PTPRF	CCL4	SDC1	TYK2	GALP	NCOA1	CDKN1B
ADARB1	CTSC	EHD1	CPS1	DEFB1	FGG	G6PD	GJA5	PCSK1N	GPR12
GSTT1	HMGCS1	HTR1B	APOBEC1	MAPT	KITLG	MYC	MYOD1	MYOG	NCF2
NDUFB6	ATP4B	OTC	OXCT1	ATP5F1D	PDE3A	PRKCD	PRKCI	PLSCR3	GPAM
RSC1A1	ABCG5	SFTPB	SFTPA1	SLC9A3	SLC10A1	AACS	BNIP3	SRD5A1	STAT4
C5AR1	EHMT1	CAV2	FADD	DMD	BEST1	APPL1	TFAP2B	EGF	ARG1
NRXN3	ATP6AP2	DUSP6	CADM2	MAP2K5	RAP1A	KISS1R	ABCA4	SETD2	HLADRB1
SOX6	MTMR9	TBX1	CEL	LINGO2	EMD	ANOS1	NF2	WWOX	WNT4

AHI1	PROK2	KMT2D	CUL4B	ELN	ARL13B	FTSJ1	GATA4	PAK3	TNNI3K
RPTOR	RFC2	BLK	SOX2	SOX3	HESX1	ARNT2	MED12	NR2E3	ZGLP1
KCNJ18	BCDIN3D-AS1	FPGT-TNNI3K	HUWE1	LINC01524	SCN1A-AS1	TOPORS	APC2	SEMA3A	MERTK
LRRC53	PRPF8	FARS2	LINC02465	LINC02055	PNPLA6	MID2	CRYZL2PSEC16B	IL1RAPL1	PNKP
ADCY9	RAB39B	CLCN4	CANT1	CNGB1	CNGA1	PROKR2	ZNF513	POC5	HGSNAT
PTCHD1	CRX	SOCS2AS1	RDH12	CTSH	C8orf37	DGKG	DCC	LRRN4	ARX
DLG3	AGTR2	ACSL4	FAT1	JMJD1C	FGF8	FHL1	CNKSR2	ZNF365	SNRNP200
IQSEC2	FLII	TTC28	SYNE1	ARHGEF18	ADNP	CRB1	ARL2BP	FLRT3	PRPF6
GABPA	GABRA3	GABRD	FSCN2	SIN3A	NSMF	COPD	PRPF31	MYCBP	TBL2
GDI1	HS6ST3	RNU1-1	RBMX	AMY1A	AMY1B	AMY1C	GNAT2	GP1BB	TTC28AS1
CCDC141	GRN	GTF2I	GUCA1B	SH3KBP1	HCFC1	HLA-DQB1	HOXB3	IDH3A	IDH3B
EYS	ZNF81	APP	APRT	KLK3	IL1RN	IMPDH1	ACADVL	ITPR3	CERKL
LHFPL3	PCARE	FEZF1	USP27X	LIMK1	EML6	LINC01122	CASC15	ARL3	HOXBAS3
SMAD4	MAK	ARVCF	MFAP1	MOG	NCAM2	RERE	NEK2	NINJ1	NRL
OTX2	P2RY11	PAX5	IMPG2	PCDH9	NIN	PDE4D	PDE6A	PDE6G	PDGFB
PDE6B	SUFU	PIK3CB	PIK3CD	PIK3CG	PLG	POU3F4	UGT1A10	UGT1A8	UGT1A7
UGT1A6	UGT1A5	UGT1A9	UGT1A4	UGT1A1	UGT1A3	IL17RD	CNNM2	POMGNT1	CHD7
WDR11	ERMARD	SPATA7	KIZ	ANGPTL8	MAPK1	KLHL7	MRPS22	HACE1	KIAA1549
SLC7A14	SUGP1	HAMP	PTX3	RAP1B	RBP3	PRPH2	RGR	RHO	RLBP1
AGBL5	ROM1	RP9	RP1	RP2	RPGR	RPE65	RPS6KA3	RREB1	SAG
CXorf56	SEMA4A	NSD1	SHOX	SKI	UPF3B	SLC5A2	SLC8A1	SMARCB1	SOX10
SRY	SYP	TACR3	TAF1	TBCE	TCOF1	TSPAN7	HIRA	TNFSF4	UFD1
USH2A	CLRN1	KDM6A	CLIP2	ZNF711	ZNF41	CA4	PRCD	CACNA1S	TMEM43
ZNF408	ALG13	RABEP2	DHDDS	GLRA3	SPG11	ALPK1	IFT88	SPRY4	USP9X
FAM161A	CCDC77	KLF11	SLC9A7	OFD1	KCNAB2	SHANK3	FGF17	PROM1	BAZ1B

AIP	PRPF4	PRPF3	CDHR1	LRAT	REEP6	HS6ST1	ARHGEF6	GTF2IRD1	SEC24C
IFT140	FRMPD4	DHX38	TRIM66	FGF19	ZBTB7C	ARSA	MPO	NNMT	BAAT
SORT1	CIDEC	SPARC	SPX	IL33	CMKLR1	GPBAR1	GRK2	PHB	CHDH
AZGP1	ACKR3	SFRP5	SI	CASR	CD59				

Table S4. The key targets

Key targets (144)									
TLR4	FAAH	SCD	IKBKB	F2	PLG	TNF	ALB	ESR1	HMGCR
NR3C1	SHBG	ERBB2	AMY1A	AMY1B	AMY1C	APP	ARG1	SERPINE1	MPO
FABP3	PRKCB	INSR	BCHE	FABP1	CA3	IGF1R	SERPINA6	NR3C2	MMP3
CYP3A4	F7	STS	ADRA2A	HMOX1	CTSH	PARP1	AR	BCL2	PTPRF
MAPT	HSPA5	FGFR1	G6PD	VDR	CYP19A1	SRC	SELL	MIF	DRD2
MMP9	BRAF	FABP4	VEGFA	SELE	IGFBP3	FLT1	PTPN1	ALOX12	SRD5A1
GABRA6	RXRA	IMPDH1	MAOA	CNR1	DRD1	DRD4	COMT	ACHE	CA4
CES1	RPS6KB1	SLC6A2	ACP1	PRKCH	EDNRA	CTSS	NPY1R	PIK3R1	HTR1B
HTR2A	HTR2C	GABRA3	MAPK1	HSD11B1	CASP1	NOS3	NOS1	TYK2	SLC5A2
SLC6A4	AKT1	CNR2	CRHR1	PTGS2	GCK	AHR	PPARG	FDFT1	STAT3
HNF4A	CCR2	AKR1C3	PIK3CA	PIK3CB	MAPK8	PLA2G4A	XDH	PIK3CG	NPY2R
MTNR1B	GSK3B	RPS6KA3	NEK2	CTSC	NR1H2	IL2	HSD11B2	SLC6A3	PPARD
LIPE	PRKCD	PTPN11	PPARA	PDE4D	KCNMA1	KCNH2	NR1H3	NR1I3	NPY5R
NFE2L2	NTRK2	HIF1A	CYP1B1	PFKFB3	TNNI3K	TRPV1	GPBAR1	ESR2	NR1H4
HDAC8	FTO	DYRK1B	KDM3A						

Table S5. Quality control (RSD) and standard curves for quantitative analysis of short-chain fatty acids

Metabolism Name	Relative Standard Deviation (RSD, %)	Retention Time (RT, min)	Calibration Curve	Coefficient of relevance（r）	Linear Range(μg/mL)
Propionic acid	0.253866535	5.48	Y = 0.01029*x + 0.001299	0.9916	0.02-500.0
Isobutyric acid	1.164188463	5.87	Y = 0.01587*x - 0.0002605	0.9986	0.02-500.0
Acetic acid	0.376346148	4.45	Y = 0.005499*x + 0.004205	0.9994	0.02-500.0
Butyric acid	0.267816774	6.73	Y = 0.03461*x + 0.001247	0.9996	0.02-500.0
Isovaleric acid	0.247376161	7.38	Y = 0.03989*x + 0.0009817	0.9995	0.02-500.0
Valeric acid	0.400432629	8.48	Y = 0.0421*x + 0.0004714	0.9995	0.02-500.0
Caproic acid	9.498348477	9.52	Y = 0.09811*x + 0.002924	0.9909	0.02-500.0

RSD < 15 % indicates good data, r > 0.99.

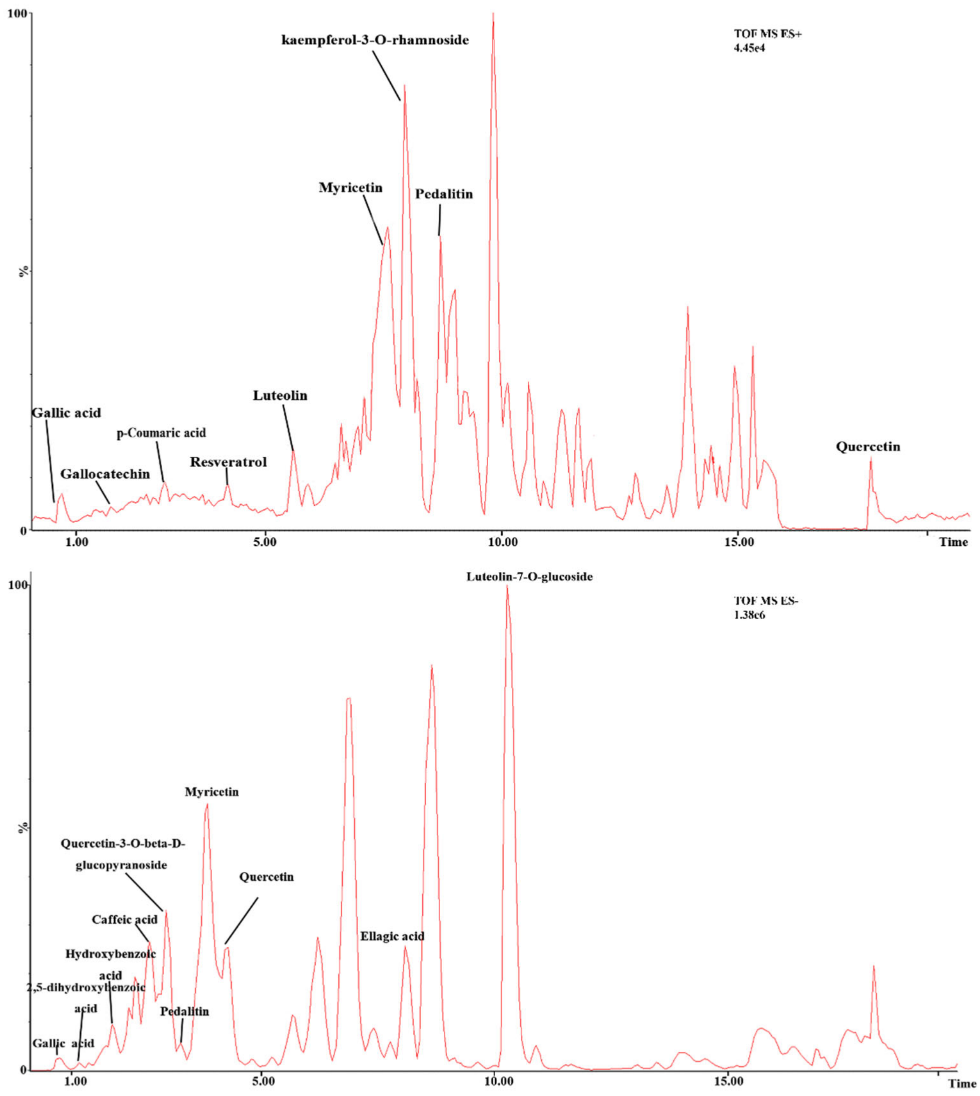

Figure S1. The relative content of the main components in hazel leaf polyphenols.

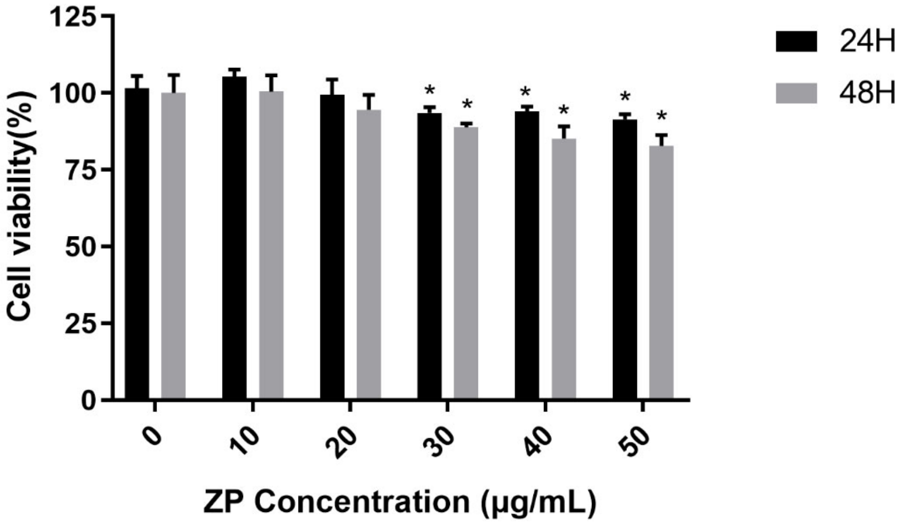

Figure S2. Evaluation of 3T3-L1 viability using MTT assay after exposure to different concentrations of hazel leaf polyphenols for 24 h or 48h, data are the mean ± SD of three replicates, and statistically significant differences versus control are denoted by**P*< 0.05.

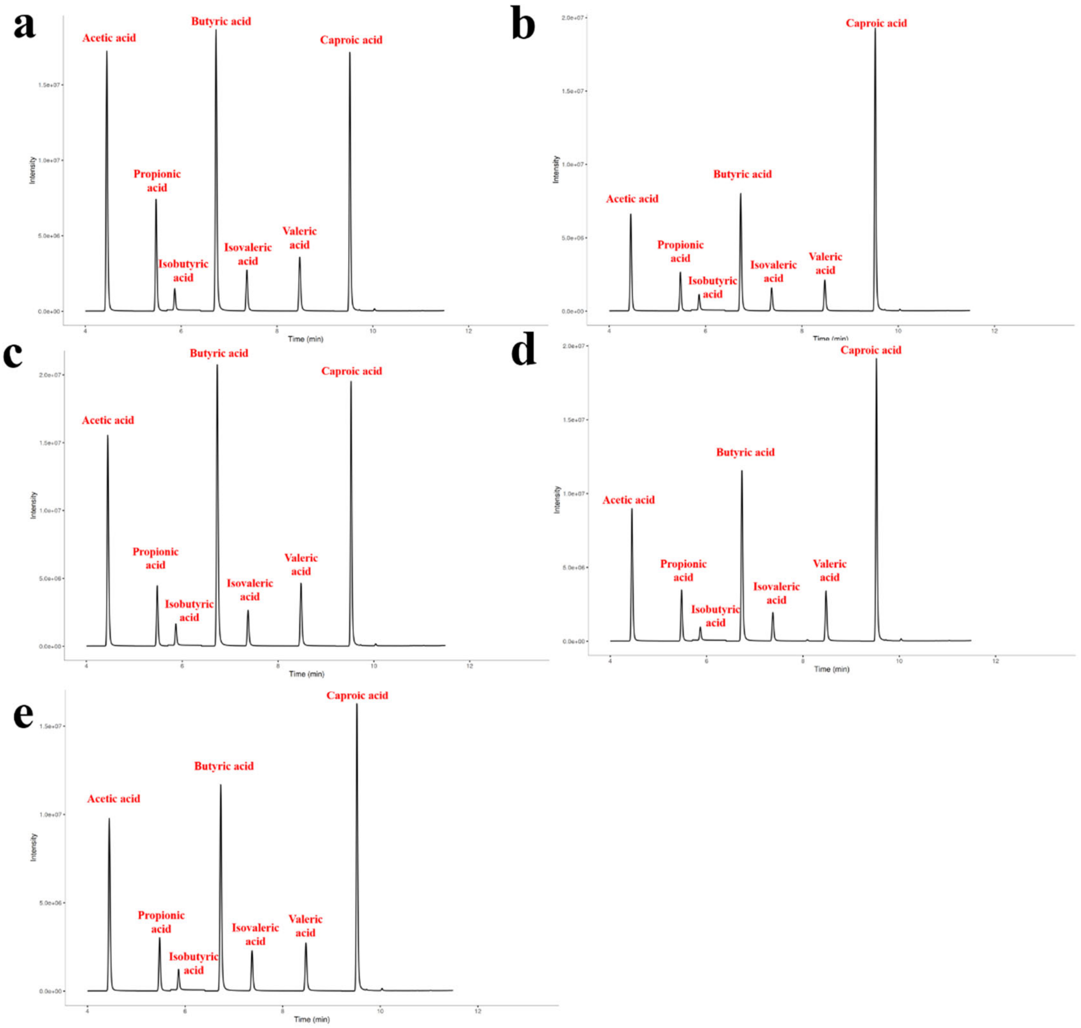

Figure S3. Total ion flow chromatograms (TIC) of different groups of short-chain fatty acids. (a) Control group. (b) Model group. (c) ZPH group. (d)ZPM group. (e) ZPL group.
